# A Nonproprietary Movement Analysis System (MoJoXlab) Based on Wearable Inertial Measurement Units Applicable to Healthy Participants and Those With Anterior Cruciate Ligament Reconstruction Across a Range of Complex Tasks: Validation Study

**DOI:** 10.2196/17872

**Published:** 2020-06-16

**Authors:** Riasat Islam, Mohamed Bennasar, Kevin Nicholas, Kate Button, Simon Holland, Paul Mulholland, Blaine Price, Mohammad Al-Amri

**Affiliations:** 1 School of Computing and Communications The Open University Milton Keynes United Kingdom; 2 Biomechanics and Bioengineering Research Centre Versus Arthritis Cardiff University Cardiff United Kingdom; 3 Cardiff and Vale University Health Board Cardiff United Kingdom; 4 School of Healthcare Sciences College of Biomedical and Life Sciences Cardiff University Cardiff United Kingdom; 5 Knowledge Media Institute The Open University Milton Keynes United Kingdom

**Keywords:** gait, algorithms, motion trackers, lower extremity, wearable electronic devices, exercise therapy, digital physiotherapy, kinematics, wearables, range of motion, anterior cruciate ligament

## Abstract

**Background:**

Movement analysis in a clinical setting is frequently restricted to observational methods to inform clinical decision making, which has limited accuracy. Fixed-site, optical, expensive movement analysis laboratories provide *gold standard* kinematic measurements; however, they are rarely accessed for routine clinical use. Wearable inertial measurement units (IMUs) have been demonstrated as comparable, inexpensive, and portable movement analysis toolkits. MoJoXlab has therefore been developed to work with generic wearable IMUs. However, before using MoJoXlab in clinical practice, there is a need to establish its validity in participants with and without knee conditions across a range of tasks with varying complexity.

**Objective:**

This paper aimed to present the validation of MoJoXlab software for using generic wearable IMUs for calculating hip, knee, and ankle joint angle measurements in the sagittal, frontal, and transverse planes for walking, squatting, and jumping in healthy participants and those with anterior cruciate ligament (ACL) reconstruction.

**Methods:**

Movement data were collected from 27 healthy participants and 20 participants with ACL reconstruction. In each case, the participants wore seven MTw2 IMUs (Xsens Technologies) to monitor their movement in walking, jumping, and squatting tasks. The hip, knee, and ankle joint angles were calculated in the sagittal, frontal, and transverse planes using two different software packages: Xsens’ validated proprietary MVN Analyze and MoJoXlab. The results were validated by comparing the generated waveforms, cross-correlation (CC), and normalized root mean square error (NRMSE) values.

**Results:**

Across all joints and activities, for data of both healthy and ACL reconstruction participants, the CC and NRMSE values for the sagittal plane are 0.99 (SD 0.01) and 0.042 (SD 0.025); 0.88 (SD 0.048) and 0.18 (SD 0.078) for the frontal plane; and 0.85 (SD 0.027) and 0.23 (SD 0.065) for the transverse plane (hip and knee joints only). On comparing the results from the two different software systems, the sagittal plane was very highly correlated, with frontal and transverse planes showing strong correlation.

**Conclusions:**

This study demonstrates that nonproprietary software such as MoJoXlab can accurately calculate joint angles for movement analysis applications comparable with proprietary software for walking, squatting, and jumping in healthy individuals and those following ACL reconstruction. MoJoXlab can be used with generic wearable IMUs that can provide clinicians accurate objective data when assessing patients’ movement, even when changes are too small to be observed visually. The availability of easy-to-setup, nonproprietary software for calibration, data collection, and joint angle calculation has the potential to increase the adoption of wearable IMU sensors in clinical practice, as well as in free living conditions, and may provide wider access to accurate, objective assessment of patients’ progress over time.

## Introduction

Within biomechanics, sports science, and physiotherapy, assessment of movement patterns for activities such as walking is vital to their practice to inform decision making around performance, recovery, and risk of reinjury [1]. In clinical settings, physiotherapy practice at present relies extensively on the visual assessment of movement quality and on the use of associated subjective clinical scales [2]. Both play important roles in decision making for treatment selection. However, there can be considerable variation in the quality of assessments depending on the experience of physiotherapists, and interrater agreement is not always as strong as expected [2]. An objective and more accurate assessment during physiotherapy sessions has the potential to facilitate more accurate diagnoses and more consistent treatment selection [3]. It also has the potential to provide objective feedback to surgeons to demonstrate the actual postoperative effect of different decisions made during surgery [4]. This is very important for patients with anterior cruciate ligament (ACL) reconstruction. ACL rupture is a common sporting injury to the knee that frequently results in surgery to reconstruct the ligament [5,6]. This is followed by a lengthy period of rehabilitation, and the ability of these individuals to return to sports varies and has been reported to be as low as 65% returning to their preinjury level of activity [7]. The reasons for this are multifactorial, but an important factor is that people with ACL reconstruction are known to move with biomechanical compensation strategies despite rehabilitation [8-10]. This can put them at risk of reinjury and future osteoarthritis, so it is important that clinicians have tools available to them in the clinical setting to assess the biomechanics during tasks that mimic sporting maneuvers [10-12].

3D motion capture camera–based systems can provide a gold standard assessment of body movement; however, such laboratories are expensive, time consuming, labor intensive, and effectively nonportable [13]. Data analysis is similarly resource intensive, time consuming, and requires specially trained personnel [14]. These limit 3D motion capture camera–based systems to research settings and are scarce in clinical practice [1]. However, wearable inertial measurement units (IMUs) can offer a similar objective assessment of body movement and are relatively much less expensive, easier to setup, mobile, and usable by clinicians with minimal training. IMU sensors consist of a triaxial accelerometer, a triaxial gyroscope, and a triaxial magnetometer. Data collected by an IMU is processed to calculate the sensor position, speed, and orientation. For certain IMUs and software, these results have been shown to be comparable to 3D motion capture camera–based systems [3]. These characteristics strongly suggest that sensors have great potential for use in clinical practice. The availability of validated and low-cost nonproprietary systems could make such systems affordable and much more widely used in clinical practice.

Existing systems pose a limitation of having complex calibration processes. Hullfish et al [2] attempted to address this issue by presenting a self-calibrated wearable sensor system for knee joint angle measurements only. Even though they have used a single, low-cost wearable inertial sensor and a simple calibration process, the system is not suitable for more complex activities such as walking, squatting, and jumping. Moreover, they have not demonstrated the use of the system in a clinical setting or included any patients. Similarly, Nazarahari et al [15] proposed a calibration method using multiple wearable IMUs to reduce measurement errors due to calibration for gait kinematics. The proposed calibration method [15] is simpler than some of the existing methods; however, the calibration requires specific movements such as hip abduction and adduction to a predefined degree, which might be a challenge for people with knee conditions.

To address the limitations mentioned earlier, we have developed nonproprietary software, MoJoXlab [16], through an academic–clinical research collaboration. MoJoXlab [16] has been developed to provide a more practical system for clinical movement analysis. The software can be used with any generic wearable IMU sensor that produces orientation angles in quaternions. It employs a simple protocol for data collection and calibration to facilitate the use of wearable IMU sensors for clinical movement analysis as the users deem fit and can also be used for diagnosis and prognosis in clinical settings. MoJoXlab [16] implements an IMU-to-body calibration method [17-19]. Although previous studies [17] have explored this method during simple activities, such as walking in healthy participants, it is yet to be explored during complex activities such as squatting or jumping, which are of interest to clinicians rehabilitating people back to sports. Within the movement analysis domain, jumping is considered to be a complex activity because of its dynamic nature, such that even conventional gait measurement equipment finds it difficult to measure accurately. The data obtained from wearable IMU sensors deviate significantly from 3D motion capture camera–based data, owing to the large impact on ground contact. The proprietary software MVN Analyze (Xsens Technologies) solves this issue to a certain extent [20]. Currently, it is the only available validated software system and as a result has been used in this research as the gold standard. However, it is limited to only Xsens’ proprietary IMU hardware. Therefore, there is a need to develop a software system that can be used with any suitable IMU.

The aim of this study was to compare the hip, knee, and ankle joint angles calculated in the sagittal, frontal, and transverse planes by MoJoXlab [16] against MVN Analyze (Xsens Technologies) from movement data collected using wearable IMU sensors during walking, squatting, and jumping in healthy people in a nonclinical setting and people with ACL reconstruction in a clinical setting.

## Methods

### Research Participants and Setting

Ethical approval for this study was obtained from Wales Research Ethics Committee 3 (10/MRE09/28). Written informed consent was obtained before participation. A sample of healthy participants (n=27) was recruited using the following criteria: age between 18 and 60 years; healthy with no known neurological, cardiovascular, or musculoskeletal conditions. Additionally, 20 participants who underwent ACL reconstruction were recruited from physiotherapy and orthopedic knee clinics in one University Health Board using the following criteria: age between 18 and 60 years, had ACL surgery in at least one of their knees within 6 to 12 months. Participant demographics are presented in Table 1.

**Table 1:** Participant demographics.

Participants	Sample, N	Average age (years)	Gender (male, female)	Average Height (cm)	Average Weight (kg)	Knee injury (right, left)
Healthy	27	35 (SD 9)	11, 16	162 (SD 34)	72 (SD 13)	0
ACL	20	29 (SD 9)	14, 6	177 (SD 11)	84 (SD 18)	8, 12

### Experimental Protocol

Each participant underwent at least one movement analysis session, with all healthy participants returning for another session within about a week later (mean 4, SD 3 days). On each day, the measurements were repeated twice, once with a biomechanics expert putting on the sensors and performing data collection, and in the other instance, a physiotherapist performed the same. Thus, a total of 4 sessions of data were recorded for each healthy participant (day 1—experiment 1 [performed by a biomechanics expert], day 1—experiment 2 [performed by a physiotherapist]; day 2—experiment 1 [performed by a biomechanics expert]; day 2—experiment 2 [performed by a physiotherapist]). The interrater reliability was acceptable across all planes for walking and squatting joint angles and for jumping it ranged from poor to excellent [3].

Data collection for all healthy participants was carried out in the Research Center for Clinical Kinaesiology at Cardiff University. In the case of participants with ACL reconstruction, data collection took place in the clinic and was conducted by a physiotherapist. For all the participants (both healthy and people with ACL reconstruction), during the first session, anthropometric measurements were taken by a physiotherapist from the right lower limb while the participant maintained a standing posture. A total of 7 MTw2 trackers (Xsens Technologies, Enschede, Netherlands) were then placed in accordance with Xsens’ instructions [21]. MTw2 trackers were secured using elasticated Velcro straps on each upper thigh (centrally and halfway between the greater trochanter and lateral epicondyle of the knee), each lower leg (proximal medial surface of the tibia), the dorsum of each foot, and one centrally over the sacrum. Each lower limb tracker was placed between the two outermost layers of the strap and attached to the Velcro of the inner layer to secure its position and minimize any movement. The sacral tracker was placed directly over the sacrum, with the upper border of the sensor aligned centrally between the two posterior superior iliac spines. The sacral sensor was held in position with medical grade double-sided adhesive tape.

Wherever possible, all the participants (both ACL and healthy) performed 8 repetitions of each of the following 3 activities: overground walking, squatting, and vertical jumping. Some of the ACL reconstruction participants were exempted from activities that they found difficult, for example, only 7 participants performed the vertical jump. Before performing each activity, the participant was provided with a demonstration by the physiotherapist and could ask any questions. The order of the activities was randomized across participants, but consistent within participants. Each walking trial consisted of a walk in a straight line across the laboratory or clinic (approximately 8 m) at the participants’ natural pace. For healthy participants, the walking trial was repeated 5 times, and for participants with ACL reconstruction, the walking was repeated 2 times. A walking trial consisted of 8 gait cycles of walking, and similarly, one such jumping or squatting trial consisted of 8 jumps and squats. The squat depth and jump height were not measured.

### MoJoXlab

MoJoXlab [16] is a MATLAB-based (version 2018b; The MathWorks Inc) custom motion capture analysis software toolkit whose aim is to produce freely available motion capture analysis software to be used by anyone interested in generating lower limb joint kinematic waveforms using any suitable IMUs [16]. MoJoXlab [16] was used in this study to generate joint angles for different functional tasks such as walking, squatting, and jumping. The joint angles are then validated against the commercially available MVN Analyze (Xsens Technologies). The joint kinematics for MoJoXlab [16] are based on a joint coordinate system, as proposed by Grood and Suntay [18]. MoJoXlab [16] takes sensor orientation data in quaternions as input and output joint angles in degrees. The joint angles generated by this method were then compared with the joint angles generated by the proprietary MVN Analyze software (Xsens Technologies). The algorithm considers a static calibration step, where sensor data are captured for calibration purposes while the participant maintains a standing pose [17]. This calibration step was then used to calculate the joint angles for the dynamic phase of the motion.

### Calibration and Data Collection

Kinematic data were collected using the MTw2 trackers at 60 Hz, and all the trackers were connected to the computer using Wi-Fi technology. The data were recorded on a computer using the Xsens MVN Analyze system (Xsens Technologies). Before beginning the tasks, the participant was asked to stand in a static N-pose, as per the instructions in the MVN Analyze user manual [21]. This was maintained for approximately 30 seconds. At the start of this period of quiet stance, the MTw2 trackers were calibrated using the MVN Analyze software (Xsens Technologies). During this process, the software establishes the relationship between the body segment and tracker orientations [18,22]. The calibration data saved within MVN Analyze (Xsens Technologies) are proprietary and cannot be extracted. Consequently, additional static calibration data were collected by asking the participant to maintain a standardized standing posture, to be used as a static calibration dataset within our custom MoJoXlab software [16,17]. This allows raw data collected from all MTw2 trackers to be projected to one global coordinate system. Then, the data for each activity were collected using MVN Analyze software (Xsens Technologies). The purpose of using the MVN Analyze software (Xsens Technologies) was two-fold: to capture the data streamed from the trackers and to later calculate the joint angles as per MVN’s proprietary algorithm to compare the joint angles with our custom MoJoXlab software [16]. However, MoJoXlab [16] uses the raw data from the same trackers to calculate joint angles and is independent of the MVN Analyze software (Xsens Technologies).

### Data Processing

As mentioned earlier, data obtained from the trackers were saved using the MVN Analyze software (Xsens Technologies). Hip, knee, and ankle joint angle calculations were also performed using the same proprietary software. All the data generated by MVN Analyze (Xsens Technologies) were exported in mvnx file formats (MVN Analyze’s open XML data format). These files were later imported to the MATLAB software (version 2018b; The MathWorks Inc), and MoJoXlab [16] was used to extract the raw sensor data from the mvnx files to calculate another set of hip, knee, and ankle joint angles.

The joint angles were generated for each activity (walk, jump, and squat), each joint (hip, knee, and ankle), each plane of movement (sagittal, frontal, and transverse), and each side of the body (left and right). However, our custom algorithm within MoJoXlab [16] could only generate angles in the sagittal and frontal planes of the ankle joint. Positive joint angles indicate flexion, abduction, and internal rotation in the sagittal, frontal, and transverse planes, respectively.

### Data Analysis and Validation

**Figure 1:**
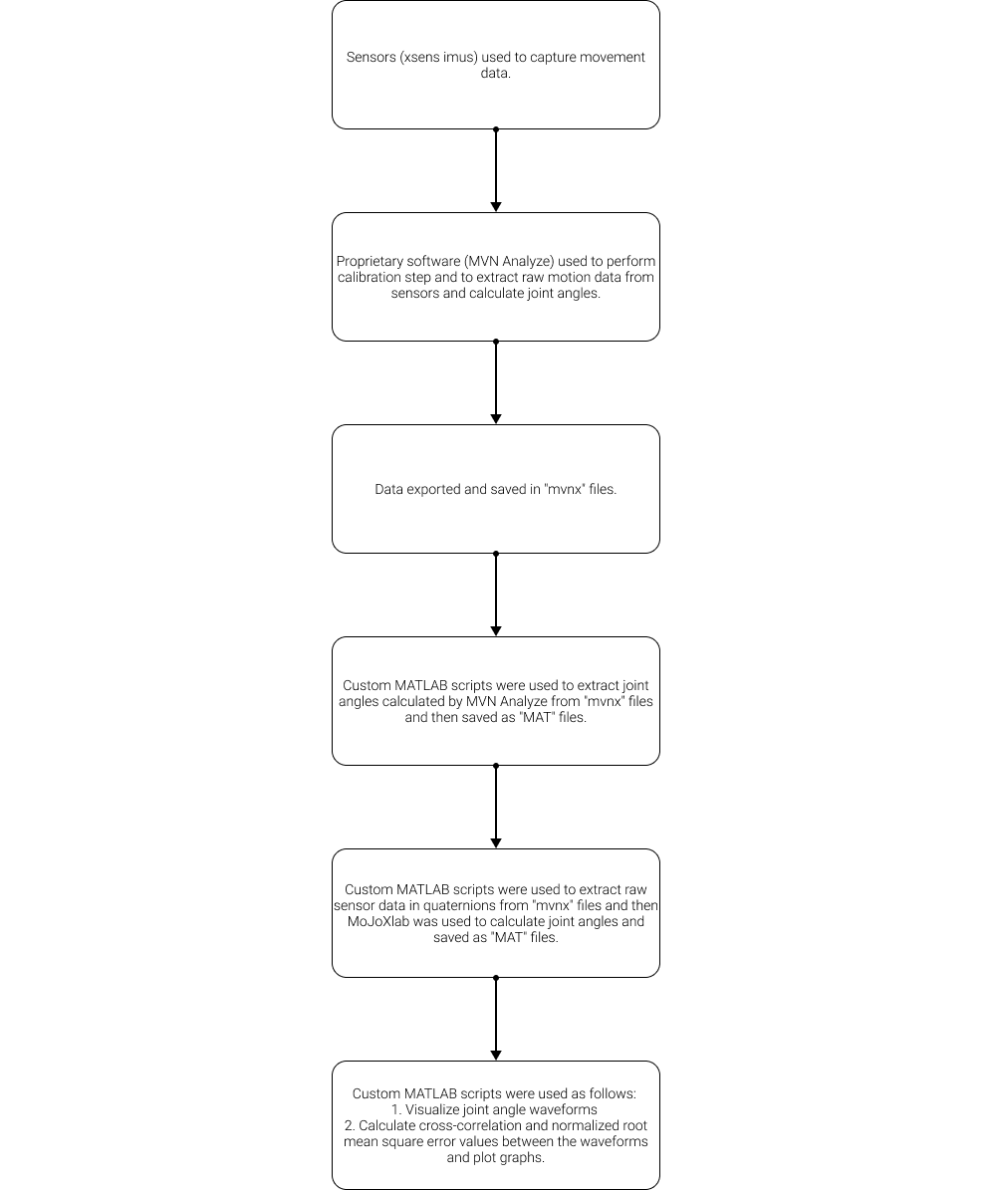
Block diagram: workflow for data processing, analysis, and validation.

Joint angles obtained by MVN Analyze (Xsens Technologies) and MoJoXlab [16] were compared and analyzed using separate custom scripts written in MATLAB (version 2018b; The MathWorks Inc). The workflow for data processing, analysis, validation, and visualization is outlined in Figure 1.

Custom MATLAB (version 2018b; The MathWorks Inc) scripts were used as follows:

1. Extract joint angles calculated by MVN Analyze (Xsens Technologies) from *mvnx* files and then saved as the MAT file (MATLAB’s data format).
2. Raw sensor data in quaternions from *mvnx* files were extracted, and MoJoXlab [16] was used to calculate another set of joint angles distinct from those calculated by MVN Analyze (Xsens Technologies). The joint angles were then saved to the MAT file.
3. Visualize joint angle waveforms and calculate cross-correlation (CC) and root mean square error (RMSE) values between the waveforms and plot their graphs.

During the data collection phase for each healthy participant, 4 trials were collected, so for 27 healthy participants, a total of 108 trials were collected. Of these, 13 were excluded from the analysis because there were some data missing for each of them. For healthy participants, 95 data trials were used for the analysis.

Similarly, for 20 ACL reconstruction participants, only one of them returned for a repeat session. All of them performed walking and squatting activities, but only 7 performed the jumping activity. Data for a total of 21 walk and squat trials were collected, and 8 jump trials were collected (1 participant performed the jump repeat session). Out of the 21 walk and squat trials, 2 were excluded as some data were missing. Thus, a total of 19 trials were used in the data analysis for the walk and squat activities, and 8 jump trials were used in the data analysis.

Waveforms of joint angles generated by MVN Analyze (Xsens Technologies) and MoJoXlab [16] were compared inside the MATLAB (version 2018b; The MathWorks Inc) environment using custom scripts, as explained earlier. In a previous study, the joint angles obtained from MVN Analyze (Xsens Technologies) were validated against joint angles obtained from the gold standard Vicon system (Vicon Motion Systems Ltd) [3]. Thus, in this study, the joint angles generated from MVN Analyze (Xsens Technologies) can be used as reference values to compare the joint angles generated by MoJoXlab [16].

#### Cross-Correlation

CC is a similarity metric used in signal processing to assess the similarity between two signals [23-25]. The resultant values are obtained as vectors. By using the *coeff* function in MATLAB (version 2018b; The MathWorks Inc), it is possible to calculate the CC coefficient between the two compared signals [26]. The metric can then be interpreted in a similar manner to the Pearson correlation coefficient, producing values between 0 and 1, with values closer to 1 indicating a higher correlation between the signals, and thus greater similarity.

CC between MoJoXlab [16] and MVN Analyze (Xsens Technologies) was calculated for each waveform to test for similarity. First, the waveforms were center normalized to have a mean of zero and corrected for polarity. The CC coefficient was calculated in the range of 0 to 1, with values closer to 1 indicating a very high correlation.

#### Normalized Root Mean Square Error

RMSE is an alternative method of measuring the differences between sets of values. In this study, RMSE was used to measure the error in joint angle values between MVN Analyze (Xsens Technologies) and MoJoXlab [16]. Two details are worth mentioning: first, applying RMSE naively to joint angles would produce error values in degrees. However, owing to the wide variety of joints, tasks, and participant groups, it would be difficult to compare RMSE values in a meaningful way across the dataset. To address this problem, the *normalized* version of the RMSE was used. This produces the values within the range of 0 to 1, where closer to 0 indicates a lower error (better agreement) between the joint angle waveforms.

Normalized root mean square error (NRMSE) was calculated to compare the joint angles between MoJoXlab [16] and MVN Analyze software (Xsens Technologies) [25,27-29]. The waveforms were standardized over the range of 0 to 1 and corrected for polarity. NRMSE was obtained within the range of 0 to 1, where values closer to 0 indicate the least difference between the waveforms.

## Results

### Waveforms

**Figure 2:**
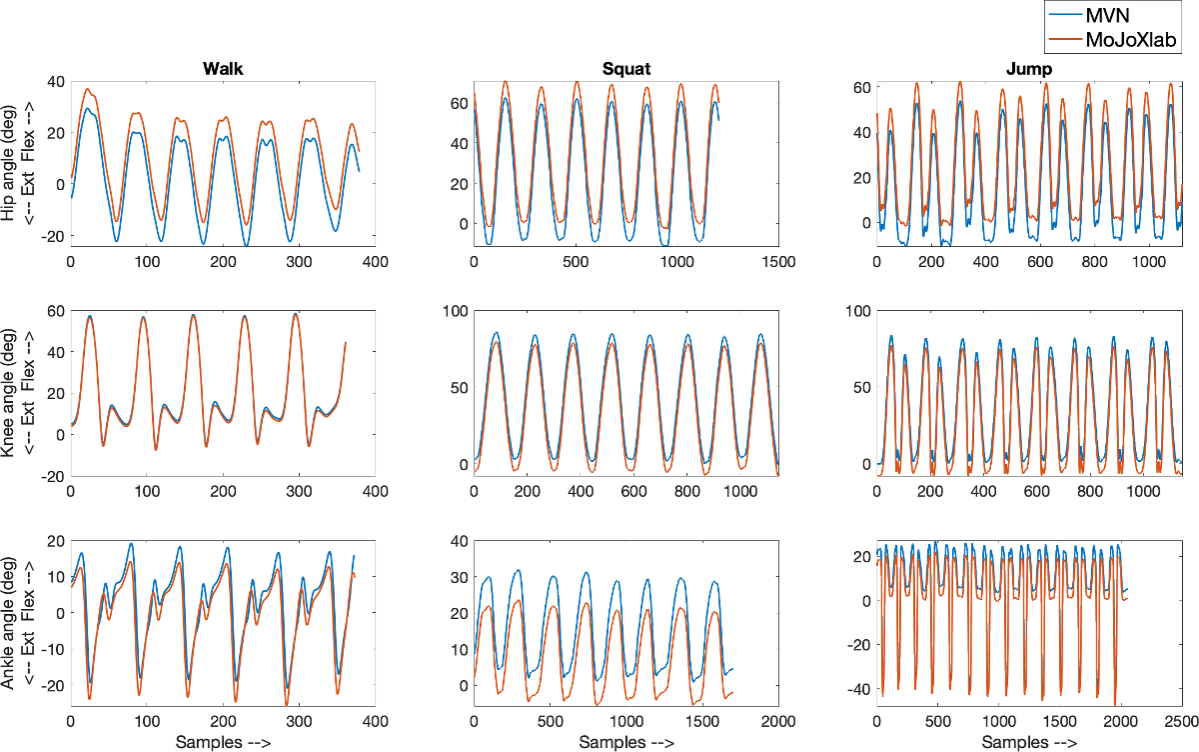
Representative sagittal plane joint angle waveforms from the healthy participant data set. Waveforms for the hip (top row), knee (middle row), and ankle (bottom row) joint angles obtained from MVN Analyze (blue) and our custom software MoJoXlab (orange) for walking (left), squatting (center), and jumping (right) tasks. The y-axis represents joint angles in degrees, and the x-axis represents data samples across the entire waveform.

**Figure 3:**
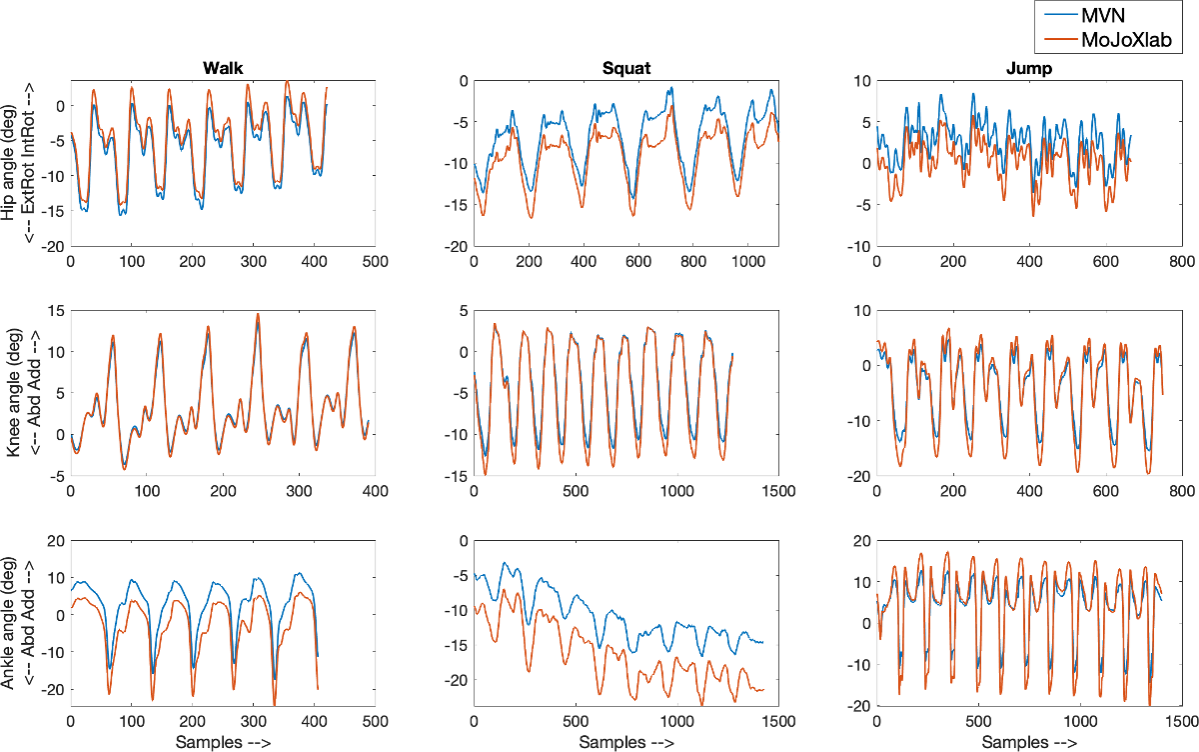
Representative frontal plane joint angle waveforms from the healthy participant data set. Waveforms for the hip (top row), knee (middle row), and ankle (bottom row) joint angles obtained from MVN Analyze (blue) and our custom software MoJoXlab (orange) for walking (left), squatting (center), and jumping (right) tasks. The y-axis represents joint angles in degrees, and the x-axis represents data samples across the entire waveform.

**Figure 4:**
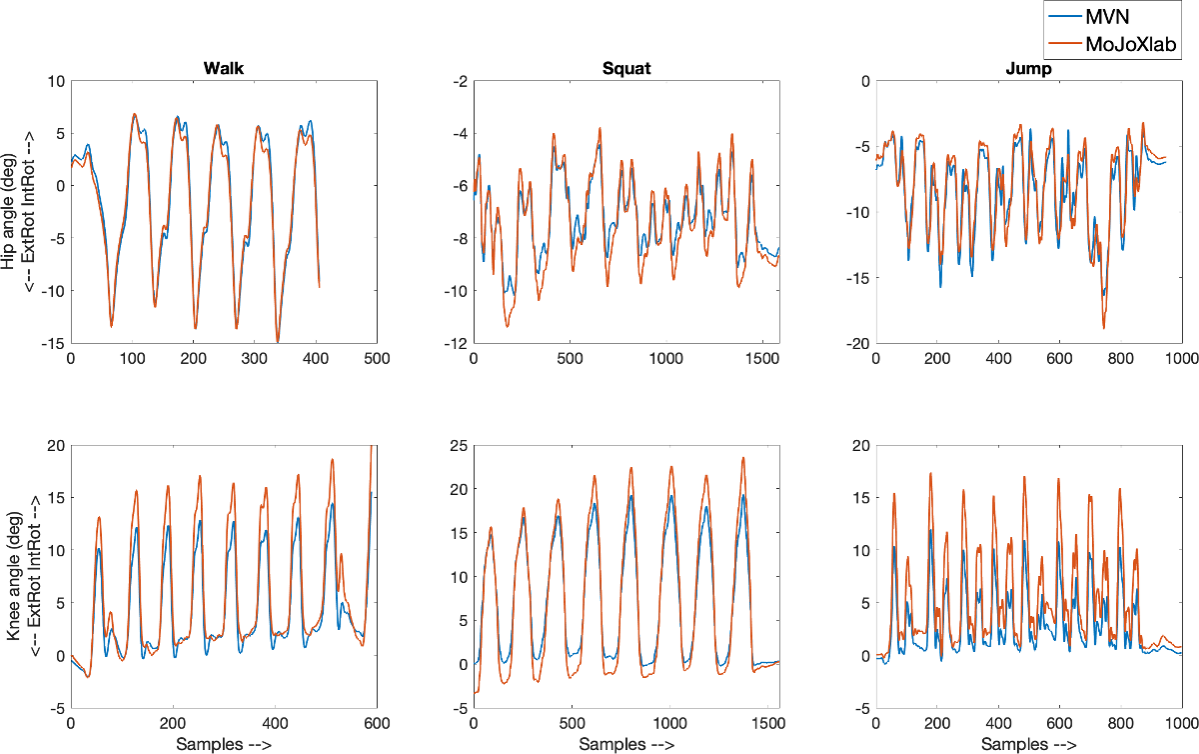
Representative transverse plane joint angle waveforms from the healthy participant data set. Waveforms for the hip (top row) and knee (bottom row) joint angles obtained from MVN Analyze (blue) and our custom software MoJoXlab (orange) for walking (left), squatting (center), and jumping (right) tasks. The y-axis represents joint angles in degrees, and the x-axis represents data samples across the entire waveform.

**Figure 5:**
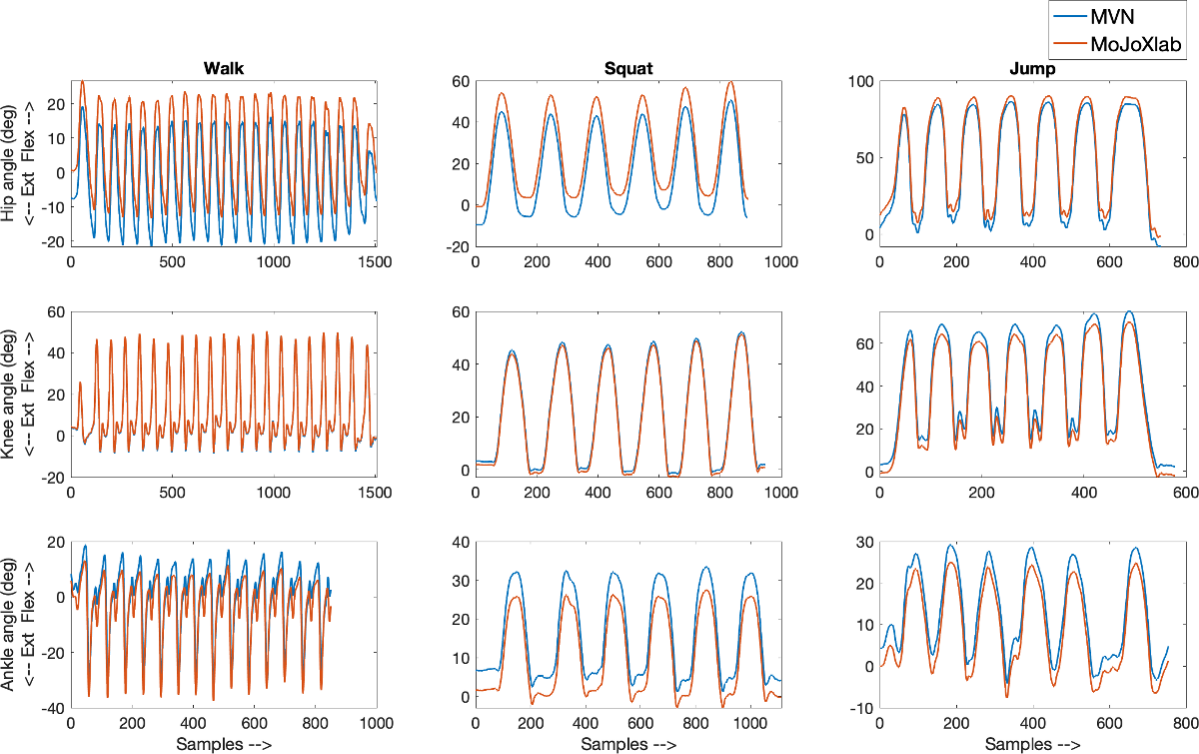
Representative sagittal plane joint angle waveforms selected from the anterior cruciate ligament reconstruction participant data set. Waveforms for the hip (top row), knee (middle row), and ankle (bottom row) joint angles obtained from MVN Analyze (blue) and our custom software MoJoXlab (orange) for walking (left), squatting (center), and jumping (right) tasks. The y-axis represents joint angles in degrees, and the x-axis represents data samples across the entire waveform.

**Figure 6:**
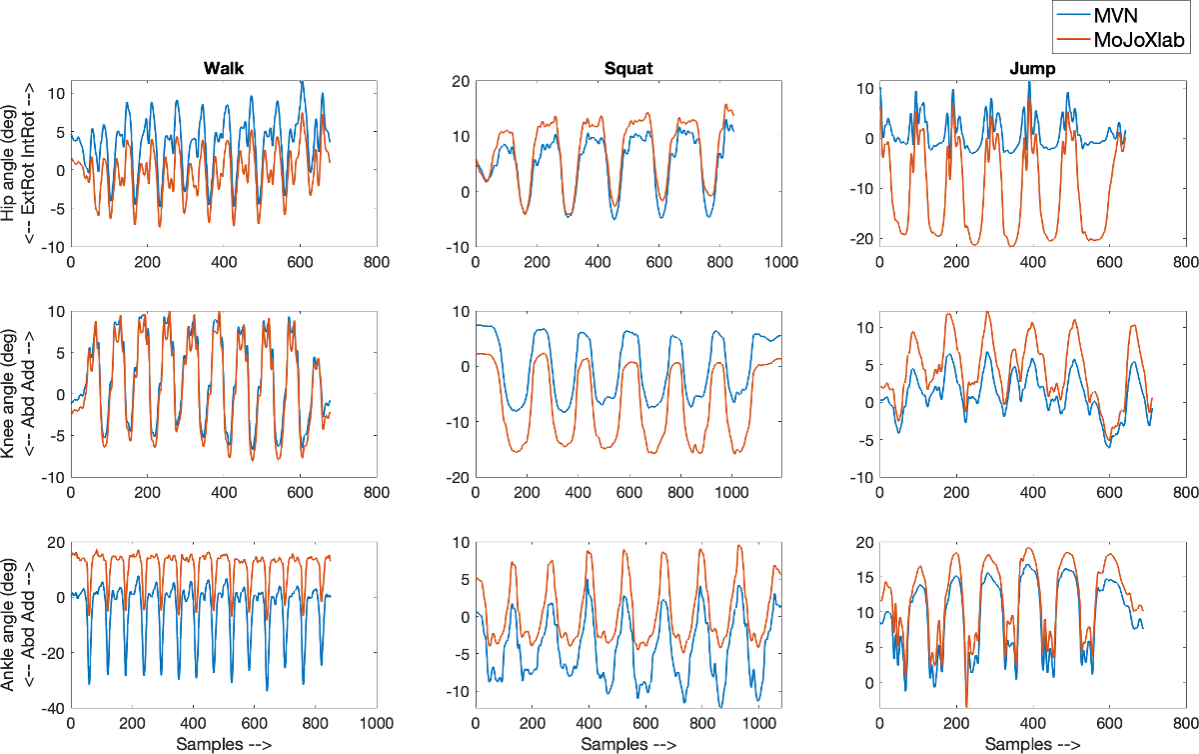
Representative frontal plane joint angle waveforms selected from the anterior cruciate ligament reconstruction participant data set. Waveforms for the hip (top row), knee (middle row), and ankle (bottom row) joint angles obtained from MVN Analyze (blue), and our custom software MoJoXlab (orange) for walking (left), squatting (center), and jumping (right) tasks. The y-axis represents joint angles in degrees, and the x-axis represents data samples across the entire waveform.

**Figure 7:**
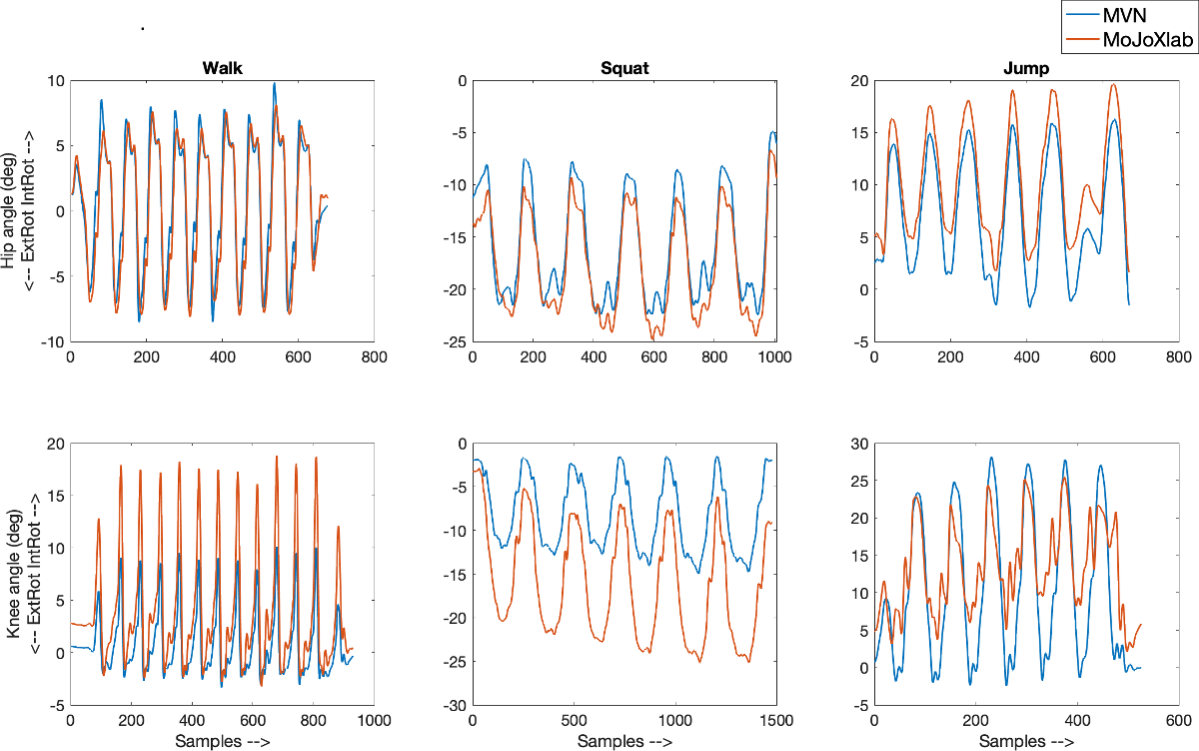
Representative transverse plane joint angle waveforms selected from anterior cruciate ligament reconstruction participant data set. Waveforms for the hip (top row) and knee (bottom row) joint angles obtained from MVN Analyze (blue) and our custom software MoJoXlab (orange) for walking (left), squat (center), and jump (right) tasks. The y-axis represents joint angles in degrees, and the x-axis represents data samples across the entire waveform.

This section presents joint angle waveforms, generated by MVN Analyze software (Xsens Technologies) and MoJoXlab [16], across all movement planes (sagittal, frontal, and transverse), for each joint (hip, knee, and ankle) and for each task (walk, squat, and jump). Figures 2-4 show representative joint angle waveforms from the healthy participant dataset. Figures 5-7 show representative joint angle waveforms from the people with ACL reconstruction dataset.

### Validation Results

This section presents the validation results for the joint angle waveforms using CC and NRMSE. The MoJoXlab [16] joint angle waveforms were compared with waveforms generated by MVN Analyze (Xsens Technologies). CC and NRMSE values were calculated for each task, each joint, and each plane. The results are presented later in parts. First, the CC values are shown for both healthy and ACL reconstruction participants. Afterward, NRMSE values are presented for healthy and ACL reconstruction participants.

The mean CC values across all participants (healthy and ACL reconstruction participants) were very high (CC>0.95) for the sagittal plane across all the joints and tasks. For healthy participants in the frontal plane across all tasks, CC>0.83, and for ACL reconstruction participants for the frontal plane across all tasks, CC>0.78. Similarly, for the transverse plane, for healthy participants across all tasks, CC>0.83, and for ACL reconstruction participants across all tasks, CC>0.84.

The NRMSE for the sagittal plane was relatively low compared with other planes for all participants (healthy and ACL across all tasks: NRMSE<0.1). For healthy participants in the frontal plane across all tasks, NRMSE<0.17, and for ACL reconstruction participants for the frontal plane across all tasks, NRMSE<0.35. Similarly, for the transverse plane, for healthy participants across all tasks, NRMSE<0.22, and for ACL reconstruction participants across all tasks, NRMSE<0.39.

In summary, for the sagittal plane across all joints and activities for both healthy and ACL reconstruction participants’ data, the CC coefficient and NRMSE are as follows: 0.99 (SD 0.01) and 0.04 (SD 0.03); similarly for the frontal plane, 0.88 (SD 0.05) and 0.18 (SD 0.08); and for transverse plane hip and knee joints only, 0.85 (SD 0.03) and 0.23 (SD 0.07), respectively.

## Discussion

### Principal Findings

This paper has demonstrated that MoJoXlab [16], our in-house developed software, can be used to calculate joint angles for movement analysis with generic wearable IMUs that report data in quaternions. MoJoXlab [16] has a simple calibration procedure, making the data collection process smooth. This makes MoJoXlab [16] potentially easier to use in clinical settings, and this paper has established its validity and demonstrated that MoJoXlab [16] can be used in a clinical setting by a clinician, across a variety of complex tasks such as walking, squatting, and jumping, and across a variety of participants, both healthy and ACL reconstruction participants. Complex tasks such as jumping are very challenging to analyze accurately with wearable IMU sensors because of the large ground impact force. MoJoXlab [16] can accurately calculate joint angles for such complex tasks and thus can be potentially extended to calculate other complex tasks and exercises as well. MoJoXlab [16] has been validated against proprietary MVN Analyze software (Xsens Technologies), which was previously validated against the VICON-based optical motion capture system (Vicon Motion Systems Ltd), considered to be clinically *gold standard* [3].

Al-Amri et al [3] concluded that joint angle waveforms obtained from MVN Analyze (Xsens Technologies) showed excellent similarity with sagittal plane waveforms obtained by the VICON system (Vicon Motion Systems Ltd) and acceptable similarity for frontal and transverse planes across all three tasks. MVN Analyze (Xsens Technologies) and VICON systems (Vicon Motion Systems Ltd) were compared using the coefficient of multiple correlation (CMC) and *R*^2^ values for the linear fit method. The CMC was found to be greater than 0.9 for all three joints in the sagittal plane across all tasks. Similarly, for the sagittal planes, the *R*^2^ value was greater than 0.8 for all the joints across all the tasks, and similarly *R*^2^ values showed fair-to-good similarity for transverse and frontal planes across all joints during squatting and jumping and knee joint during walking. Thus, by the transitive property, we claim that MoJoXlab [16] can generate joint angles comparable with optical *gold standard* motion capture systems.

In the following sections, we discuss the validation results between MoJoXlab [16] and MVN Analyze (Xsens Technologies) for each of the planes in two ways: by comparing the joint angle waveforms across CC and by computing the NRMSE. We also discuss differences in healthy participants versus ACL reconstruction participants across activities.

### Cross-Correlation

For all joints, across all tasks and participants (both ACL reconstruction participants and healthy participants), the sagittal plane shows a very high correlation, with mean CC above 0.95. This indicates that MoJoXlab [16] generates sagittal plane joint angle waveforms that are highly similar to those of MVN Analyze (Xsens Technologies). The sagittal plane reflects joint angles for flexion and extension of the joints, which are most commonly referred by clinicians [30], to assess recovery and potential risk factors for injury to the ACL. Reduced range of motion in this plane is often associated with incomplete recovery and poor neuromuscular control. For example, reduced knee flexion during landing from a jump is associated with higher peak moments at the knee joint [31].

Similarly, the frontal plane is also useful for clinicians who are interested in abduction and adduction of the joints, as this is considered a risk factor for reinjury, poor neuromuscular control, and incomplete recovery [30,32]. In the case of frontal planes, CCs are also high for all joints: with values across all tasks and participant groups for ankle joints being greater than 0.84, for hip joints being greater than 0.78, and for knee joints being greater than 0.83.

In the case of the transverse plane, MoJoXlab [16] can calculate joint angles for the hip and knee joints only. In this plane, CC values across all tasks and participant groups for the hip are greater than 0.83 and for knee joints, greater than 0.83.

**Figure 8:**
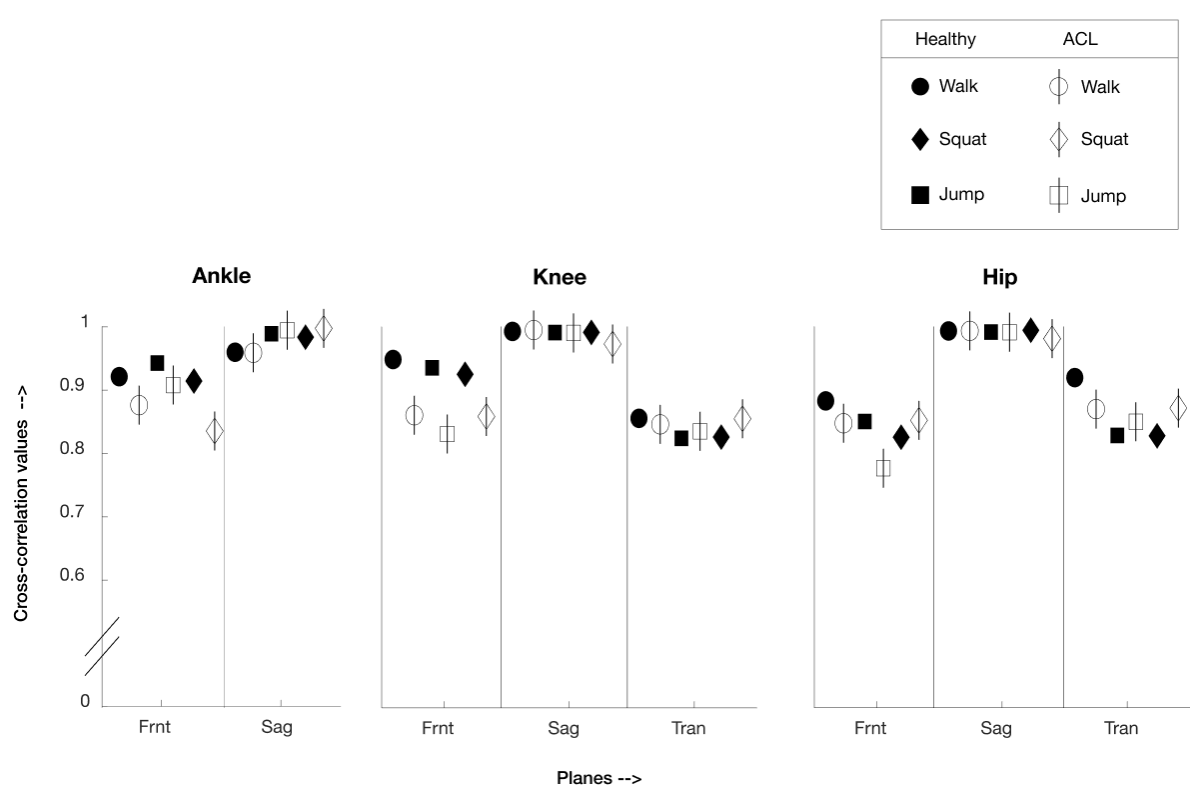
Mean cross-correlation values between MVN Analyze and MoJoXlab for healthy participants (black) and anterior cruciate ligament reconstruction participants (white), presented in a graphical format for visualization and overall comparison purposes. Values close to 1 indicate a very high correlation (circle—values for the walk task, square—values for jump task, and diamond—values for squat task; planes: Frnt—frontal, Sag—sagittal, and Tran—transverse).

Overall, by observing the representative waveforms (Figures 2-7) and the high CC values (Figure 8), it is evident that MoJoXlab [16] software can produce joint angles comparable with the commercial MVN Analyze software (Xsens Technologies).

Previous studies comparing software to calculate joint angles using wearable IMUs are limited. Hullfish et al [2] investigated knee joint angles in the sagittal plane only for seven healthy participants. They compared their IMUs with an optical motion capture system. Their CC values were within the range of 0.84 to 0.99. In comparison with these values, we obtained a mean CC range of greater than 0.95 for the sagittal plane across all participant groups, activities, and all joints. For other planes, the CC is generally greater than 0.83 except for the frontal plane for ACL reconstruction participants, where the values are greater than 0.78.

These results are comparable with those of previous studies and further extend previous work in healthy participants, which reported high agreement between joint angle waveforms in the sagittal plane for systems using IMUs and an optical motion capture system [33,34]. Other studies have compared data obtained from Xsens IMUs for walking [35,36], squatting [14,37], and jumping [38,39]. However, our results extend previous work by including more challenging dynamic tasks such as squatting and jumping. We also evaluated the validity of software in people with ACL reconstruction in addition to healthy people. The results confirm that MoJoXlab software [16] can be used to assess tasks such as squatting and jumping in healthy individuals and individuals following ACL reconstruction within a clinical setting.

### Normalized Root Mean Square Error

**Figure 9:**
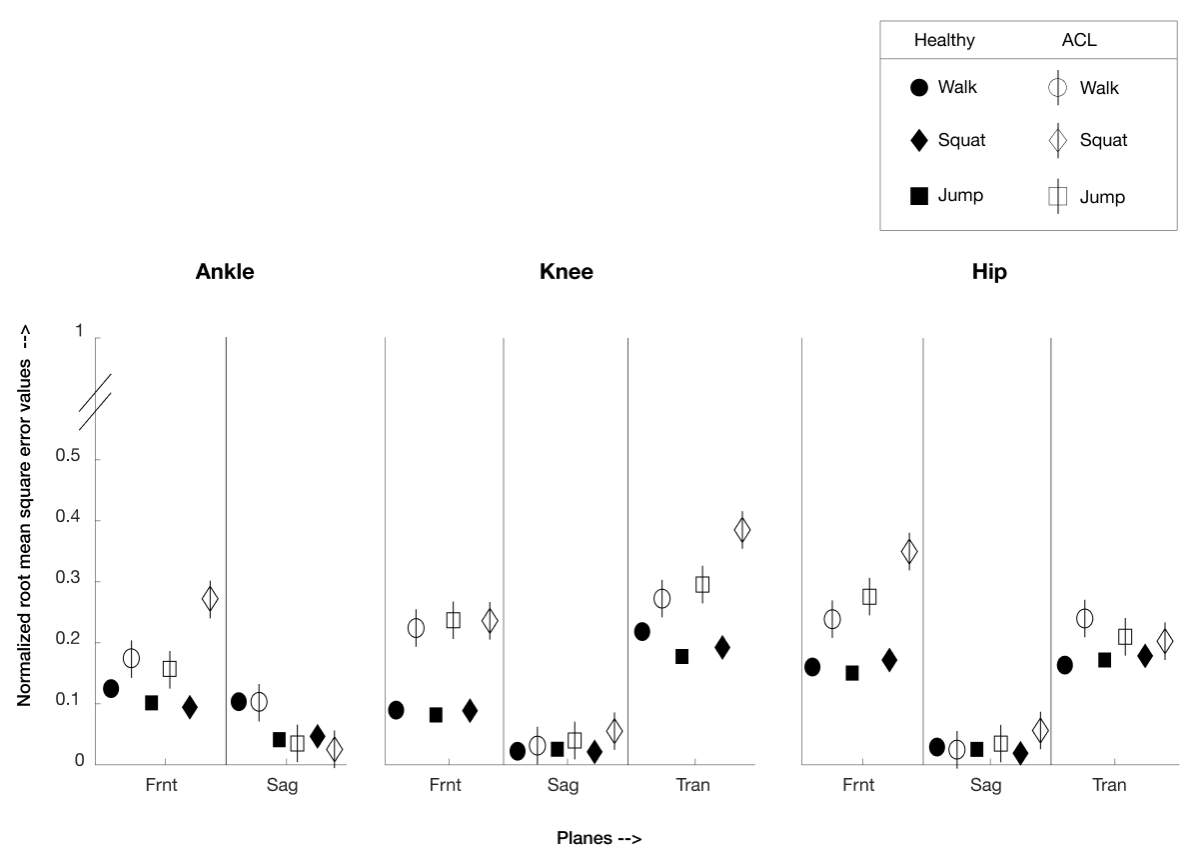
Normalized root mean square error values between MVN Analyze and MoJoXlab for healthy participants (black) and ACL reconstruction participants (white), presented in a graphical format for visualization and overall comparison purposes. Values close to 0 indicate a very low error (circle—values for the walk task, square—values for jump task, and diamond—values for squat task; planes: Frnt—Frontal, Sag—Sagittal, and Tran—Transverse).

CC as a measure of similarity is blind to both constant vertical offsets and differences in amplitude. It should be noted that blindness to constant vertical differences are not of material interest for the purposes of the study; however, differences in amplitude are of considerable importance because they represent entirely different joint angle ranges. For this reason, it is valuable to use a complementary measure of similarity, which is highly sensitive to differences in amplitude. In particular, the RMSE corresponds to a single number representing the Pythagorean distance in a high-dimensional space between the two waveforms and is highly sensitive to differences in amplitude, frequency, and offset. To allow meaningful comparison of RMSE values between different activities and joints, we have given the results as NRMSE, where the RMSE is divided in each case by the range (Figure 9).

As noted previously, CC values for sagittal planes showed very high agreement between the waveforms generated by the two systems. Similarly, the NRMSE values obtained for sagittal planes also showed a very low error (NRMSE<0.1) across all tasks, joint angles, and participant groups. The low NRMSE values in conjunction with very high CC values suggest that MoJoXlab [16] can generate joint angle waveforms in the sagittal plane that are highly comparable with commercially available MVN Analyze software (Xsens Technologies).

In the case of frontal planes, the healthy participant joint angles showed lower error values (NRMSE<0.17) than the ACL reconstruction participants group (NRMSE<0.35). Similarly, in the transverse plane, the healthy participant joint angles showed lower error values (NRMSE<0.22) than the ACL reconstruction participants group (NRMSE<0.39). Thus, the NRMSE values for the ACL reconstruction participants group for both the frontal and transverse planes were higher than their respective healthy participant group values. The error values for joint angles for the frontal and transverse planes for healthy participants are within the reasonably accepted range of 0.2. The high CC and low NRMSE values for all healthy participants across all tasks and joints suggest excellent agreement between MoJoXlab [16] and MVN Analyze (Xsens Technologies).

In summary, in the case of the ACL reconstruction participant group, values for the sagittal plane show high CC and low NRMSE values, suggesting excellent agreement. For ACL reconstruction participants, in the transverse and frontal planes, the CC values are high, thus confirming agreement on waveform pattern similarities between MoJoXlab [16] and MVN Analyze (Xsens Technologies). However, the underlying reasons behind the slightly higher range of NRMSE error values for the ACL reconstruction participants group for frontal planes and transverse planes requires further investigation.

### Understanding the Differences in Waveforms

The differences in joint angle waveforms for the same task and joint noted above may be due to several possible contributing factors. One of the significant contributing factors is the static calibration step described in the Methods section. The calibration step carried out by the proprietary MVN system produces no externally inspectable data that can be used in this study. The calibration values captured by MVN during the calibration step were saved internally within the software. The values are not accessible to the user either on the software interface or when all the data are exported as *.mvnx files. Thus, MoJoXlab [16] does not have access to MVN calibration values. A separate set of values were captured for MoJoXlab [16] as its calibration step, while the participant maintained the same standing posture. In principle, the calibration values should be similar to the data being captured for the same standing posture. However, it is reasonably possible that minuscule movements can vary the calibration values, even more so for ACL reconstruction participants than for healthy participants. It is likely that ACL reconstruction participants might find it difficult to maintain the same standing posture while the calibration steps are carried out. This might be a contributing factor to the difference in waveforms between the two software systems and also for the slightly larger NRMSE values or the ACL reconstruction participants in comparison with healthy participants. In contrast, the healthy participants might have held similar static postures for the static calibration step, resulting in similar calibration values feeding to the two software systems. As a result, the waveforms are more in agreement for this participant group.

Another potential contributing factor is the different sites for data collection. For the healthy participant group, both the static calibration steps and the functional tasks were measured in the laboratory. However, in the ACL reconstruction participants group, the calibration step was undertaken in the consultation room within the clinic, and some of the activities took place outside of the consultation room in the corridor or in a different room. The different sites for data collection for some of the tasks can account for the difference in the waveforms as a number of external factors can contribute to the difference in waveforms between the two software systems. One such external factor is the presence of equipment in the clinic that causes magnetic interference. The physiotherapist conducting the data collection in the clinic noted that there was external magnetic interference affecting the sensor data. In principle, as the two software packages use the same raw sensor data, it can be understood that magnetic interference should not affect the outcome of the joint angle waveforms. However, there can be a difference in waveforms because MoJoXlab [16] and MVN Analyze (Xsens Technologies) handle magnetic interference in different ways. Currently, MoJoXlab [16] does not have any special software or algorithm that handles magnetic interference from the environment, and this is a limitation of the current version of MoJoXlab [16]. However, Xsens claims that they have special software in MVN Analyze to handle magnetic interference from the environment, even though such claims have not yet been validated [40]. To use MoJoXlab [16] with data collected in clinical settings, people should be careful to determine whether magnetic interference severely affects the data. It is possible that discrepancies could occur when collecting data over time due to external magnetic interference [41].

In clinical settings, 3D motion capture camera–based systems are generally used for movement analysis, which tracks markers attached to the body over a certain field of space. As a result, it is possible to detect the movement of the body frame and body segments across time and space. However, motion tracking using IMUs uses an inherently different principle, where the relative angular motion of each IMU sensor is combined using sensor fusion algorithms to calculate the joint angles for the hip, knee, and ankle joints. Thus, one of the limitations of using IMUs for clinical movement analysis is that joint angles are considered separately for each joint; thus, phenomena such as *shifting* of the knee cannot be detected by simply considering joint angles [42].

One of the major limitations of this study is that, while the number of trials available for analysis in the healthy participant group was quite large at 96, the number was relatively small for ACL reconstruction participants, with walking and squatting tasks having 19 available trials, whereas the jumping task had only eight available trials. This disparity in the number of trials available for data analysis between healthy and ACL reconstruction participants can also be one of the contributing factors to the difference in results. Further work is required to collect more data from people with ACL reconstruction for a better comparison between healthy and ACL reconstruction participants’ data.

### Further Work

MoJoXlab [16] is currently under development in collaboration with Cardiff University and the Open University. This paper presented only the validation results between the waveforms generated by MoJoXlab [16] and the proprietary MVN software. Further work is required to validate the various gait parameters calculated by MoJoXlab [16] and to enable MoJoXlab [16] to better handle external factors that can affect the data, such as magnetic interference. Although MoJoXlab [16] can work with any sensor that reports quaternions, in this particular study, the IMU data were collected using Xsens’ wearable IMU sensors. In the future, we would like to test how different wearable IMU sensors can be used with MoJoXlab [16].

### Conclusions

This study has shown that a variety of clinically relevant functional tasks such as walking, squatting, and jumping can be measured using wearable IMUs in both laboratory and clinical settings, by clinicians (in this case physiotherapists) using nonproprietary software. We have developed and validated this nonproprietary software against software that has been shown to be as accurate as an optical motion capture system. Validation results suggest that MoJoXlab [16] can calculate joint angles comparable with proprietary MVN Analyze software (Xsens Technologies) across people with ACL reconstruction and healthy people, for tasks such as walking and more complex tasks such as squatting and jumping. Thus, MoJoXlab [16] has the potential to provide clinicians with accurate movement analysis of their patients across multiple joints and planes of motion and may be able to provide an analysis of other complex tasks such as lunging and jumping. These reflect advanced rehabilitation and sporting maneuvers that individuals following ACL reconstruction (and other injuries) need to be able to return to. It can potentially enable clinicians to benefit from using generic wearable IMUs in their practice to capture movement data of their clients and objectively track changes over time. Increasing the adoption of such software and sensors in clinical practice has the potential for better decision making around exercise prescription, monitoring patient progress over time, tailoring advice and feedback, and improving the rehabilitation process.

## Abbreviations

ACL: anterior cruciate ligament
CC: cross-correlation
CMC: coefficient of multiple correlation
IMU: inertial measurement unit
NRMSE: normalized root mean square error
RMSE: root mean square error
